# 典型和不典型免疫表型慢性淋巴细胞白血病遗传学和分子生物学特征研究

**DOI:** 10.3760/cma.j.issn.0253-2727.2022.06.005

**Published:** 2022-06

**Authors:** 慧敏 金, 纯 乔, 四书 赵, 海荣 仇, 肖 陈, 慧 杨, 莉颖 朱, 建勇 李, 雨洁 吴

**Affiliations:** 南京医科大学第一附属医院（江苏省人民医院）血液科，南京 210029 The First Affiliated Hospital of Nanjing Medical University, Jiangsu Province Hospital, Hematology Department, Nanjing 210029, China

**Keywords:** 白血病，淋巴细胞，慢性, 免疫表型分型, 细胞遗传学, 分子生物学, Leukemia, lymphocytic, chronic, Immunophenotyping, Cytogenetics, Molecular biology

## Abstract

**目的:**

探讨典型和不典型免疫表型慢性淋巴细胞白血病（CLL）在免疫表型、遗传学及分子生物学方面的差异及遗传学异常与基因突变的相关性。

**方法:**

依据英国马斯登皇家医院免疫分型积分系统对2014年11月至2021年5月期间南京医科大学第一附属医院收治的488例初诊CLL患者进行分类，其中积分4～5分为典型免疫表型CLL（tCLL）（382例），积分3分为不典型免疫表型CLL（aCLL）（106例）。采用多参数流式细胞术对所有CLL患者外周血标本进行免疫表型检测，荧光原位杂交（FISH）技术检测359例CLL患者的遗传学异常，二代测序（NGS）技术检测330例CLL患者基因突变情况。

**结果:**

aCLL患者CD10、CD22、CD49d、CD81和FMC7阳性表达率显著高于tCLL患者（*P*值分别为0.020、<0.001、<0.001、0.027和<0.001），CD5、CD23、CD148和CD200的阳性表达率显著低于tCLL患者（*P*值分别为<0.001、0.017、0.041和<0.001）。aCLL患者+12阳性率显著高于tCLL患者（*P*<0.001），del（13q14）阳性率显著低于tCLL患者（*P*<0.001），同时aCLL患者NOTCH1突变发生率高于tCLL患者（*P*＝0.038），其余基因突变发生率在两组间的差异均无统计学意义（*P*值均>0.05）。生存分析显示，tCLL和aCLL患者总生存（OS）率与无治疗生存（TFS）率的差异无统计学意义（*P*值均>0.05）。

**结论:**

tCLL与aCLL患者的抗原表达特征、细胞遗传学及体细胞突变均存在差异，有助于tCLL与aCLL的诊断和鉴别诊断。

## Abstract (en)

**Objective:**

To analyze the differences in immunophenotype, cytogenetics, and molecular biology between typical and atypical immunophenotype chronic lymphocytic leukemia (CLL), and explore the correlation of cytogenetic anomalies with gene mutations.

**Methods:**

This study included 488 patients diagnosed in the First Affiliated Hospital of Nanjing Medical University between November 2014 and May 2021. Of these, 382 patients scored 4–5 points, which was typical CLL (tCLL), and 106 scored 3 points, which was atypical CLL (aCLL) as per the Royal Marsden Hospital Immunomarker Integral System. Peripheral blood cells were collected for immunophenotype by multiparameter flow cytometry in 488 patients, fluorescence in situ hybridization (FISH) was employed to detect cytogenetic anomalies in 359 patients, and gene mutations were detected by next-generation sequencing (NGS) in 330 patients.

**Results:**

The positive rates of CD10, CD22, CD49d, CD81, and FMC7 were significantly higher in the aCLL compared with the tCLL group (*P*＝0.020, *P*<0.001, *P*<0.001, *P*＝0.027, and *P*<0.001, respectively), while the positive rates of CD5, CD23, CD148, and CD200 were lower in the former compared to the latter (*P*<0.001, *P*＝0.017, *P*＝0.041, and *P*<0.001, respectively). aCLL exhibited a higher frequency of trisomy 12 and lower frequency of del (13q14) compared to the tCLL group (*P*<0.001 and *P*<0.001, respectively). Moreover, aCLL patients also showed a higher incidence of NOTCH1 mutations than the tCLL patients (*P*＝0.038), while no statistically significant differences in other gene mutations occurred between the two groups. No significant differences in overall survival (OS) and treatment-free survival (TFS) occurred between aCLL and tCLL using Kaplan-Meier analysis (*P*>0.05).

**Conclusion:**

aCLL has characteristic immunophenotype, cytogenetic, and somatic mutation that differ from tCLL, and this can provide reliable information for the diagnosis and differential diagnosis between the two groups.

慢性淋巴细胞白血病（chronic lymphocytic leukemia，CLL）是一种淋巴细胞克隆性增殖的恶性血液病，具有典型的形态学和免疫学表型特征。但随着对CLL研究的深入，发现CLL在疾病诊断、发病形式、治疗反应及预后等方面有很大异质性。NCCN指南提出，典型的CLL免疫表型为CD19^+^CD5^+^CD23^+^CD10^−^CD20^dim^slg^dim^，但也存在小部分以slg^bright^或CD23^−/dim^或CD20^bright^为特征的不典型免疫表型。越来越多的学者对具有不典型免疫表型的CLL进行研究。有研究根据Moreau Score（MS）积分系统，将积分<4或CD23^−^定义为不典型免疫表型CLL（atypical chronic lymphocytic leukemia，aCLL）[1]。与典型免疫表型CLL（typical chronic lymphocytic leukemia，tCLL）相比，aCLL患者WBC和淋巴细胞绝对计数更高，而PLT较低[2]。推测tCLL和aCLL的发生发展和转归可能存在异质性。近年来，随着二代测序（next generation sequencing, NGS）技术逐渐应用于临床，发现了参与CLL发生发展的众多基因突变，涉及NOTCH信号通路、B细胞受体信号通路、细胞凋亡、表观遗传学调控、炎症因子通路、DNA损伤修复与细胞周期调控、RNA代谢等。目前研究多集中于aCLL与tCLL在形态学、免疫表型及遗传学等方面的研究，而分子生物学相关研究尚无报道。本研究回顾性分析488例CLL患者的免疫表型特征，探索aCLL和tCLL细胞遗传学和基因突变的差异、相关性及患者预后的差异。

## 病例与方法

1. 病例：收集2014年11月至2021年5月南京医科大学第一附属医院（江苏省人民医院）血液科收治的488例初诊CLL患者的临床资料。根据英国马斯登皇家医院（RMH）免疫标志积分系统，积分4～5分为tCLL（382例），积分3分为aCLL（106例）。男330例（67.6％），女158例（32.4％），中位年龄61（22～88）岁，所有患者均符合CLL诊断标准[3]。积分3分病例常规采用荧光原位杂交（FISH）检测t（11;14），免疫组织化学（IHC）检测CyclinD1、SOX11，均为阴性后排除套细胞淋巴瘤（mantle cell lymphoma, MCL）；通过组织病理学和FISH检测t（14;18）排除滤泡淋巴瘤（follicular lymphoma, FL）。一些具有不典型形态的淋巴浆细胞性淋巴瘤（lymphoplasmacytic lymphoma, LPL）多为CD23^−^，结合MYD88 L265P的突变位点等特征进行综合判断并排除。极少数CD5^+^边缘区B细胞淋巴瘤（marginal zone B-cell lymphoma, MZL）通常表现为CD23^−^CD200^−^，结合临床和组织病理学特点排除。根据形态学特点和诊断标准排除CD5^+^幼稚淋巴细胞白血病（prolymphocytic leukemia, PLL）。

2. 流式细胞术（FCM）免疫分型：应用多参数FCM分析CLL细胞表面不同抗原的表达情况（美国Beckman Navios流式细胞仪）。应用的单克隆靶向抗体包括：CD5（ECD）、CD10（PE）、CD19（A750）、CD148（PE）、CD20（PC7）、CD22（PB）、CD200（APC）、CD79b（PC5.5）、CD23（APC）、IgM（PB）、CD45（KO）、CD49d（PE）、FMC7（FITC）、CD35（FITC）、CD38（ECD）、CD25（PC5.5）、CD11c（PC7）、CD103（APC）、CD81（FITC）、CD160（PE）、CD19（PC5.5）、Kappa（FITC）、Lambda（PE）（美国Beckman公司）。具体检测步骤参照文献[4]，应用Kaluza软件分析免疫表型特点，抗原表达率以≥20％为阳性。

3. 染色体核型分析和FISH：共485例CLL进行染色体核型分析；共359例CLL采用GLP D13S319、LSI TP53探针、LSI MBY 探针、LSI ATM DNA探针、CEP +12 DNA探针、LSI IGH探针（美国Vysis公司），运用I-FISH技术分别检测del（13q14）、del（17p13）、del（6q23）、del（11q23）、+12和IGH重排，于Olympus BX60荧光显微镜下观察间期细胞荧光杂交信号，具体步骤详见说明书。以上细胞遗传学异常分别以大于10％、5％、8％、8％、5％、8％为阳性标准。

4. 分子生物学：采用IGVH基因引物和IGH体细胞超突变凝胶检测分析试剂盒（InVivoScribe公司）检测IGVH基因突变，具体参照文献[5]。应用IMGT/V-QUEST数据库对测序结果进行比对分析，以VH碱基突变率≥2％作为体细胞突变标准。

收集患者初诊时骨髓单个核细胞1.0×10^7^，采用DNA提取试剂盒（北京TIANGEN公司）提取基因组DNA，以至少100 ng DNA进行扩增建库，应用Illumina MiSeq测序仪对40个基因外显子热点区域测序，平均测序深度1000×，使用TMAP（torrent mapping alignment program）软件分析测序结果。检测基因包括：ARID1A、ATP6AP1、ATP6V1B2、ATM、BIRC3、BRAF、BTK、BCL2、CARD11、CDKN1B、CHD2、CXCR4、CCND1、CREBBP、EGR2、EP300、EZH2、FOXO1、FAT1、FBXW7、IGLL5、KMT2D、KLF2、KRAS、MAP2K1、MYD88、NFKBIE、NOTCH1、NOTCH2、TP53、PLCG2、PTPRD、POT1、RPS15、RRAGC、SAMHD1、SF3B1、TET2、TNFRSF14、XPO1。

5. 统计学处理：采用SPSS 19.0进行数据分析，计数资料用例数（百分比）描述，计量资料用*M*（范围）描述。计量资料的比较采用*t*检验，率的比较采用*χ*^2^检验，总生存（OS）率与无治疗生存（TFS）率采用Kaplan-Meier法进行生存分析，采用Log-rank检验进行生存率比较，所有统计学分析采用双侧检验，*P*<0.05为差异有统计学意义。

## 结果

1. 临床特征：488例CLL患者中，aCLL 106例（21.7％），tCLL 382例（78.3％）。aCLL患者的中位年龄为65（33～88）岁，tCLL患者的中位年龄为59（22～86）岁。与aCLL患者相比，tCLL患者男性比例较高（*P*＝0.023），≥60岁比例较低（*P*＝0.047），HGB较高（*P*＝0.031）。两组患者WBC、淋巴细胞绝对计数、PLT、β_2_-微球蛋白（β_2_-MG）、LDH及Binet分期等的差异均无统计学意义（*P*值均>0.05）（表1）。

**表1:** aCLL与tCLL患者的临床特征比较

临床特征	aCLL（106例）	tCLL（382例）	*χ*^2^值/*t*值	*P*值
性别［例（％）］			5.158	0.023
男	62（58.5）	268（70.2）		
女	44（41.5）	114（29.8）		
年龄［例（％）］			3.949	0.047
<60岁	42（39.6）	193（50.5）		
≥60岁	64（60.4）	189（49.5）		
WBC［×10^9^/L，*M*（范围）］	23.8（3.3～690.9）	23.3（1.0～540.4）	0.846	0.398
ALC［×10^9^/L，*M*（范围）］	19.1（0.2～594.9）	18.4（0.6～392.4）	0.829	0.408
HGB［g/L，*M*（范围）］	116.0（45.0～182.0）	123.0（36.0～170.0）	−2.159	0.031
PLT［×10^9^/L，*M*（范围）］	121.0（9.0～451.0）	131.0（2.0～365.0）	−0.225	0.822
β_2_-微球蛋白［mg/L，*M*（范围）］	3.6（1.0～19.0）	3.4（1.3～15.8）	1.203	0.230
LDH［mg/L，*M*（范围）］	216.0（114.0～1574.0）	212.0（110.0～1092.0）	1.401	0.162
Binet分期［例（％）］			2.581	0.275
A期	30（31.6）	108（29.4）		
B期	23（24.2）	120（32.6）		
C期	42（44.2）	140（38.0）		
治疗方案［例（％）］			1.307	0.520
传统免疫化疗^a^	25（52.1）	94（43.7）		
新药单药^b^	7（14.6）	43（20.0）		
联合用药^c^	16（33.3）	78（36.3）		

注：aCLL：不典型免疫表型慢性淋巴细胞白血病；tCLL：典型免疫表型慢性淋巴细胞白血病；ALC：淋巴细胞绝对计数；LDH：乳酸脱氢酶；^a^ 以嘌呤类似物或苯丁酸氮芥为基础的化学免疫治疗等；^b^ BTK抑制剂或Bcl-2抑制剂等；^c^ 以嘌呤类似物或苯丁酸氮芥为基础的化学免疫治疗联合BTK抑制剂或Bcl-2抑制剂

2. 免疫表型特征：488例aCLL和tCLL患者进行了23个抗原表达分析，aCLL患者CD10、CD22、CD49d、CD81和FMC7的阳性表达率显著高于tCLL患者（*P*值分别为0.020、<0.001、<0.001、0.027和<0.001），而aCLL患者CD5、CD23、CD148和CD200的阳性表达率显著低于tCLL（*P*值分别为<0.001、0.017、0.041和<0.001）（图1）。

**图1:**
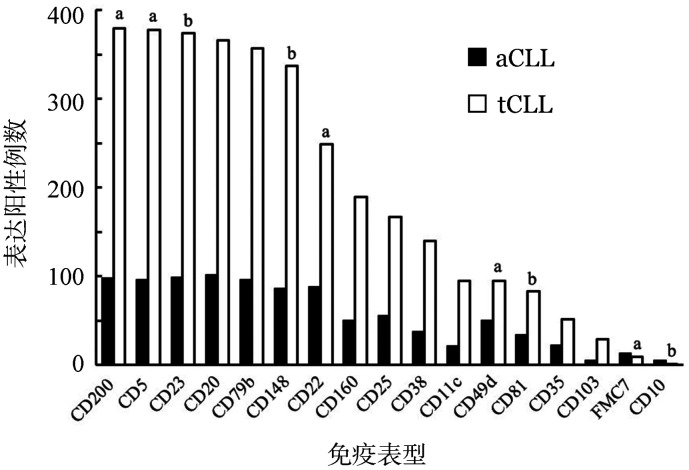
aCLL与tCLL免疫表型特征比较 aCLL：不典型免疫表型慢性淋巴细胞白血病；tCLL：典型免疫表型慢性淋巴细胞白血病；与aCLL相比，^a^ *P*<0.001，^b^ *P*<0.05

3. 遗传学特征：359例患者进行FISH检测，aCLL中+12及IGH重排阳性率显著高于tCLL组（*P*<0.001和*P*＝0.002），而aCLL中del（13q14）阳性率显著低于tCLL组（*P*<0.001）。两组del（17p13）、del（11q23）及del（6q23）的阳性表达率差异均无统计学意义（*P*>0.05）。485例患者进行染色体核型分析，22例aCLL（20.8％）和89例tCLL（23.5％）存在复杂核型（CP）异常（*P*>0.05）（表2）。

**表2:** aCLL与tCLL患者分子遗传学特征比较［例（％）］

组别	del(17p13)阳性	del(11q23)阳性	del(13q14)阳性	+12阳性	del(6q23)阳性	IGH重排阳性	复杂核型	IGHV突变
aCLL	5（7.1）	8（11.4）	15（21.4）	25（35.7）	5（7.1）	18（25.7）	22（20.8）	41（50.6）
tCLL	38（13.1）	57（19.7）	149（51.6）	35（12.1）	10（3.5）	32（11.1）	89（23.5）	154（49.4）

*χ*^2^值	1.882	2.615	20.614	22.554	1.890	9.746	0.349	0.041
*P*值	0.218	0.106	<0.001	<0.001	0.184	0.002	0.554	0.840

注：aCLL：不典型免疫表型慢性淋巴细胞白血病；tCLL：典型免疫表型慢性淋巴细胞白血病

4. 分子生物学特征：81例aCLL患者和312例tCLL患者检测IGHV突变，两组突变率的差异无统计学意义（*P*＝0.840）。采用NGS对330例初诊CLL患者（aCLL 78例，tCLL 252例）进行40个基因外显子热点区域测序。aCLL基因突变率为53.8％（42/78），其中5.1％（4/78）携带三个及以上基因突变，突变频率最高的基因依次为NOTCH1（15.4％）、SF3B1（10.3％）和IGLL5（7.7％）（图2）。tCLL基因突变率为62.7％（158/252），其中8.7％（22/252）携带三个及以上基因突变，突变频率最高的基因依次为ATM（11.1％）、TP53（9.5％）和NOTCH1（7.5％）（图2）。aCLL组的NOTCH1突变率显著高于tCLL组（*P*＝0.038），两组间其余基因突变率的差异均无统计学意义（*P*值均>0.05）。进一步选取突变发生率最高的6个基因，分析其基因突变与遗传学异常及IGHV突变状态是否存在关联。结果显示，aCLL中，SF3B1和NOTCH1突变与IGHV无突变型相关（*P*值分别为0.027和0.008）（图3A）。tCLL中，TP53突变和NOTCH1突变与IGHV无突变型相关（*P*值分别为0.017和<0.001）（图3B）。aCLL和tCLL患者IGLL5突变均与IGHV突变型相关（*P*值分别为0.018和<0.001），两组患者中TP53突变在del（17p13）异常的患者中更为常见（图3）。在aCLL中，SF3B1突变与NOTCH1突变相关（*P*＝0.017）（图3A）。

**图2:**
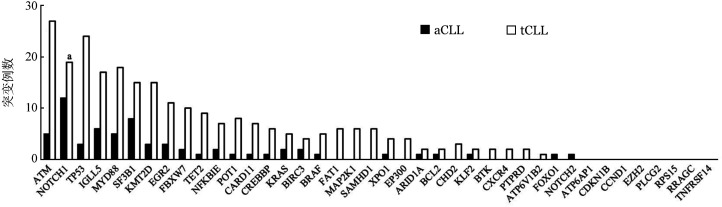
aCLL与tCLL患者基因突变分布图 所有基因仅列出一级变异。aCLL：不典型免疫表型慢性淋巴细胞白血病；tCLL：典型免疫表型慢性淋巴细胞白血病；与aCLL相比，^a^ *P*＝0.038

**图3:**
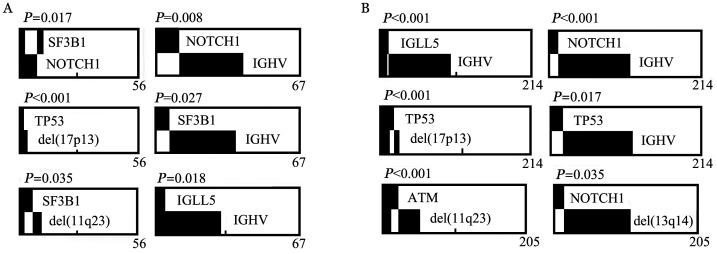
aCLL（A）与tCLL（B）患者基因突变与分子遗传学异常的相关性 黑色表示阳性或突变，白色表示阴性或无突变，横坐标表示纳入统计的例数。aCLL：不典型免疫表型慢性淋巴细胞白血病；tCLL：典型免疫表型慢性淋巴细胞白血病

5. 生存分析：随访时间截至2021年7月1日，79例aCLL随访患者中，有48例（60.8％）患者因出现治疗指征（参照国际CLL工作组指南判定）而接受治疗，其中15例患者诊断时即满足治疗指征，33例在观察等待过程中出现治疗指征，另外31例（39.2％）患者随访期内未接受治疗，8例因感染或疾病进展等死亡。整组患者的中位无治疗生存（TFS）时间为38.0（95％*CI* 31.8～44.2）个月，中位OS时间为54.0（95％*CI* 46.0～62.0）个月。337例tCLL随访患者中，有215例（63.8％）患者因出现治疗指征（参照国际CLL工作组指南判定）接受治疗，其中62例患者诊断时即满足治疗指征，153例患者在观察等待过程中出现治疗指征，另外122例（36.2％）患者随访期内未接受治疗，28例因感染或疾病进展等死亡。整组患者的中位TFS时间为58.9（95％*CI* 38.8～79.0）个月，中位OS时间为73.9（95％*CI* 49.2～98.6）个月。根据治疗方案进一步将aCLL和tCLL分别分为传统免疫化疗组、新药单药组、联合用药组，对其进行生存分析，差异均无统计学意义（*P*值均>0.05）。在传统免疫化疗组中，aCLL患者的中位OS时间为52.5（95％*CI* 45.9～59.1）个月，低于tCLL患者的中位OS时间59.0（95％*CI* 58.0～60.0）个月，差异无统计学意义（*P*＝0.059）。

## 讨论

既往CLL诊断主要依赖细胞形态学，FAB组织基于形态学特征将CLL分成三种亚型：经典型、幼稚淋巴细胞白血病（外周血涂片中有10％～55％幼稚淋巴细胞）及不典型形态的CLL（特征是细胞形态比经典型更大，细胞质更丰富或带有裂隙的淋巴细胞）。资料显示，具有不典型形态学的CLL发生率为10.0％～36.0％[6]，本中心aCLL占21.7％。随着多参数FCM应用于CLL的诊断，如今主要通过免疫表型特征分析诊断CLL。本组病例结果显示，aCLL中最常见的表型特征为CD5、CD23阴性及FMC7阳性，与文献[7]–[8]报道一致。Ting等[9]发现，aCLL中CD200阳性表达率显著高于MCL，本研究显示aCLL中CD200和CD148的阳性表达率显著低于tCLL，同时发现CD10、CD22、CD49d和CD81阳性表达率显著高于tCLL，因此这些免疫标记可以对两者进行鉴别诊断。虽然有学者认为在aCLL中CD38高表达[10]，但本研究中两组患者CD38阳性表达率的差异无统计学意义。

CLL常伴随的del（17p）、del（11q）、del（13q）、+12等细胞遗传学异常参与了疾病的发生、发展。文献报道，与tCLL相比，aCLL+12异常比例更高[6],[10]，而tCLL仅伴del（13q）异常的患者比例明显高于aCLL[11]。本研究发现，aCLL中有25例（35.7％）患者存在+12异常，显著高于tCLL（*P*<0.001），del（13q）异常发生率显著低于tCLL（*P*<0.001）。有学者发现，+12与较差的预后相关，del（13q）异常多存在于tCLL且与良好预后相关[10]。

近年来，高通量测序技术的发展极大地促进了CLL分子异常的研究，NOTCH1和SF3B1被认为是最常发生于CLL的分子突变。本研究中突变频率最高的基因是ATM，占10.0％，其次是NOTCH1（9.4％）和TP53（8.2％），与文献报道的初诊CLL中ATM、NOTCH1、TP53的突变率10.0％～20.0％、4.7％～12.3％、4.0％～10.0％一致[12]–[14]。NOTCH1基因位于9号染色体长臂，NOTCH1突变导致其蛋白高表达，进而促进NOTCH1信号通路激活，参与了CLL细胞的生存、抗凋亡及耐药。有学者认为CLL细胞NOTCH1表达水平较其他类型淋巴瘤高，将NOTCH1突变纳入CLL预后不良的重要指标之一，且与Richter转化存在密切联系[15]–[17]。本研究发现，aCLL中NOTCH1突变发生率较tCLL更高，差异有统计学意义（*P*＝0.038），且NOTCH1突变与IGHV无突变型存在明显相关性。本研究提示，在aCLL或tCLL中，IGLL5突变常与IGHV突变同时发生，与Kasar等[18]得出的结论类似，此外，IGLL5突变是低风险CLL患者的独特分子特征。

综上所述，既往aCLL与tCLL的差异研究主要局限于形态学、免疫表型以及遗传学，尚无两者分子学异常差异的研究报道。我们结合NGS比较了两组患者基因突变的差异，进而探索了遗传学异常和基因突变的相关性。本研究发现，与tCLL相比，aCLL常伴随CD49d阳性、+12异常、NOTCH1突变，较少出现del（13q），这些特征不仅有助于aCLL和tCLL的鉴别诊断，同时也有助于对两者的生物学特征进行深入挖掘。我们根据用药情况对两组患者的OS时间和TFS时间进行分析，发现aCLL和tCLL的差异无统计学意义（*P*值均>0.05）。Criel等[19]的研究发现，与tCLL相比，aCLL的OS时间更短，预后更差，分析其原因可能如下：地域人群和分类方式等不同造成生存差异，本研究通过免疫表型将CLL分为aCLL和tCLL，而Criel等[19]通过形态学分类；由于新药时代来临，临床广泛使用包括BTK抑制剂、BCL-2抑制剂等靶向药物，极大地改善了CLL患者的生存率导致两者差异减小；本研究观察的时间较短。在后续研究中，我们将进一步扩大样本量并进行长期随访，深入研究aCLL和tCLL在预后方面的差异。
